# Supplementary material for: The Advanced C.A.R.E. Suicide Prevention Gatekeeper Training Questionnaire for medical lecturers and specialists: A psychometric evaluation in a Malaysian sample
Source: Glob Ment Health (Camb). 2023 Jul 25;10:e37. doi: 10.1017/gmh.2023.32 (PMC10579687; doi:10.1017/gmh.2023.32)
Supplement: Supplementary file 1 [file S2054425123000328sup001.docx]

**Supplementary File**

**Supplementary Figure 1.** The Advanced C.A.R.E. Suicide Gatekeeper Training Questionnaire After Exploratory Factor Analysis.

| **Section A** | | | | | | | | | | |
| --- | --- | --- | --- | --- | --- | --- | --- | --- | --- | --- |
| 1 | | | Date |  | | | | | | |
| 2 | | | Profession |  | | | | | | |
| 3 | | | Organization |  | | | | | | |
| 4 | | | Email |  | | | | | | |
| **Section B** | | | | | | | | |  | |
| *How much do you* ***know*** *about the following subjects?* | | | | | Very Poor Average Good Excellent  Poor | | | | | |
| 1 | | | Knowledge on suicide prevention | | 1 | 2 | 3 | 4 | 5 | |
| 2 | | | Warning signs of suicide | | 1 | 2 | 3 | 4 | 5 | |
| 3 | | | Communicating with someone who is suicidal | | 1 | 2 | 3 | 4 | 5 | |
| 4 | | | How to arrange help for a suicidal person | | 1 | 2 | 3 | 4 | 5 | |
| **Section C** | | | | | | | | | | |
| *How far do you agree with the following statements:* | | | | | Strongly Disagree Neutral Agree Strongly  Disagree Agree | | | | | |
| 1 | Depression is a potential suicide risk. | | | | 1 2 3 4 5 | | | | | |
| 2 | People who are suicidal may not see a way out of their problems. | | | | 1 2 3 4 5 | | | | | |
| 3 | Acknowledging a suicidal person’s distress should be done before offering any advice. | | | | 1 2 3 4 5 | | | | | |
| 4 | A person who shows warning signs of suicide should be referred to a healthcare provider. | | | | 1 2 3 4 5 | | | | | |
| 5 | Crisis helplines should be offered to a suicidal person. | | | | 1 2 3 4 5 | | | | | |
| **Section D** | | | | | | | | | | |
| *How far do you agree with the following statements:* | | | | | Strongly Disagree Neutral Agree Strongly  Disagree Agree | | | | | |
| 1 | | | I have confidence in my abilities to recognize warning signs of suicide in people. | | 1 | 2 | 3 | 4 | 5 | |
| 2 | | | I hesitate to ask a person whether they are suicidal | | | 1 2 3 4 5 | | | | |
| 3 | | | I have confidence in my abilities to arrange for help for someone who is suicidal. | | 1 | 2 | 3 | 4 | 5 | |
| 4 | | | I am confident in discussing about safety planning with someone who is suicidal. | | 1 | 2 | 3 | 4 | 5 | |
| 5 | | | I know where to seek resources for postvention services. | | 1 | 2 | 3 | 4 | 5 | |

**Supplementary Figure 2.** The Advanced C.A.R.E. Suicide Gatekeeper Training Questionnaire Before Exploratory Factor Analysis

| **Section A** | | | | | | | | | | |
| --- | --- | --- | --- | --- | --- | --- | --- | --- | --- | --- |
| 1 | | | Date |  | | | | | | |
| 2 | | | Profession |  | | | | | | |
| 3 | | | Organization |  | | | | | | |
| 4 | | | Email |  | | | | | | |
| **Section B** | | | | | | | | |  | |
| *How much do you* ***know*** *about the following subjects?* | | | | | Very Poor Average Good Excellent  Poor | | | | | |
| 1 | | | Knowledge on suicide prevention | | 1 | 2 | 3 | 4 | 5 | |
| 2 | | | Warning signs of suicide | | 1 | 2 | 3 | 4 | 5 | |
| 3 | | | Communicating with someone who is suicidal | | 1 | 2 | 3 | 4 | 5 | |
| 4 | | | How to arrange help for a suicidal person | | 1 | 2 | 3 | 4 | 5 | |
| **Section C** | | | | | | | | | | |
| *How far do you agree with the following statements:* | | | | | Strongly Disagree Neutral Agree Strongly  Disagree Agree | | | | | |
| 1 | Most people who attempt suicide show warning signs before their attempt. | | | | 1 2 3 4 5 | | | | | |
| 2 | Suicide is usually caused by more than one factor. | | | | 1 2 3 4 5 | | | | | |
| 3 | Only people who have been diagnosed with mental illness are at risk of suicide. | | | | 1 2 3 4 5 | | | | | |
| 4 | Depression is a potential suicide risk. | | | | 1 2 3 4 5 | | | | | |
| 5 | Making final plans or giving away prized possessions are warning signs for suicide. | | | | 1 2 3 4 5 | | | | | |
| 6 | People who talk about suicide are less likely to attempt suicide. | | | | 1 2 3 4 5 | | | | | |
| 7 | Asking about suicidal thoughts will cause a person to develop suicidal ideas. | | | | 1 2 3 4 5 | | | | | |
| 8 | People who are suicidal may not see a way out of their problems. | | | | 1 2 3 4 5 | | | | | |
| 9 | Understanding a suicidal person’s method of suicide is necessary for safety planning. | | | | 1 2 3 4 5 | | | | | |
| *How far do you agree with the following statements:* | | | | | Strongly Disagree Neutral Agree Strongly  Disagree Agree | | | | | |
| 10 | Acknowledging a suicidal person’s distress should be done before offering any advice. | | | | 1 2 3 4 5 | | | | | |
| 11 | A person who shows warning signs of suicide should be referred to a healthcare provider. | | | | 1 2 3 4 5 | | | | | |
| 12 | We must never disclose a person’ s suicidal plan without their permission. | | | | 1 2 3 4 5 | | | | | |
| 13 | Crisis helplines should be offered to a suicidal person. | | | | 1 2 3 4 5 | | | | | |
| 14 | Describing explicit details about suicidal methods in the media is harmful. | | | | 1 2 3 4 5 | | | | | |
| 15 | I must consider my own safety when attending to a suicidal person. | | | | 1 2 3 4 5 | | | | | |
| **Section D** | | | | | | | | | | |
| *How far do you agree with the following statements:* | | | | | Strongly Disagree Neutral Agree Strongly  Disagree Agree | | | | | |
| 1 | | | I have confidence in my abilities to recognize warning signs of suicide in people. | | 1 | 2 | 3 | 4 | 5 | |
| 2 | | | I hesitate to ask a person whether they are suicidal | | | 1 2 3 4 5 | | | | |
| 3 | | | I have confidence in my abilities to arrange for help for someone who is suicidal. | | 1 | 2 | 3 | 4 | 5 | |
| 4 | | | I am confident in discussing about safety planning with someone who is suicidal. | | 1 | 2 | 3 | 4 | 5 | |
| 5 | | | I know where to seek resources for postvention services. | | 1 | 2 | 3 | 4 | 5 | |
